# Gene Editing Strategies for Duchenne Muscular Dystrophy: From Molecular Mechanisms to Clinical Translation

**DOI:** 10.3390/cells15100852

**Published:** 2026-05-07

**Authors:** Ayesha Siddika, Joël Rousseau, Félix Veillette, Camille Bouchard, Yaoyao Lu, Jacques P. Tremblay

**Affiliations:** 1 Département de Médecine Moléculaire, Université Laval, Québec, QC G1V 0A6, Canada; ayesha.siddika@crchudequebec.ulaval.ca (A.S.); felix.veillette.4@ulaval.ca (F.V.); camille.bouchard@crchudequebec.ulaval.ca (C.B.); yaoyao.lu@crchudequebec.ulaval.ca (Y.L.); 2 Centre de Recherche du Centre Hospitalier Universitaire de Québec, Québec, QC G1E 6W2, Canada; joel.rousseau@crchudequebec.ulaval.ca

**Keywords:** Duchenne muscular dystrophy (DMD), dystrophin, *DMD* gene, gene editing therapy, CRISPR/Cas9, base editing, prime editing, translational therapy

## Abstract

**Highlights:**

**What are the main findings?**

- This review introduces a comparative, mutation-guided framework linking gene-editing strategies (CRISPR/Cas9, base editing, and prime editing) to specific DMD mutation types rather than describing them in isolation.
- It integrates recent advances in precision editing technologies and translational evidence, highlighting how editing mechanism, efficiency, and repair outcomes influence therapeutic suitability.

**What are the implications of the main findings?**

- The study reframes gene editing as a mutation-guided therapeutic strategy, supporting the development of durable and potentially curative interventions for Duchenne muscular dystrophy.
- It identifies delivery, systemic targeting, and long-term safety as the primary barriers to clinical translation, providing a roadmap for next-generation therapeutic development.

**Abstract:**

Duchenne muscular dystrophy (DMD) remains a major challenge in genetic medicine due to the difficulty of achieving durable, body-wide restoration of dystrophin in post-mitotic muscle tissues. Although current therapies—including exon skipping and micro-dystrophin gene replacement—have demonstrated clinical feasibility, their benefits are limited by incomplete efficacy, mutation specificity, and the need for repeated or high-dose interventions. These limitations highlight the need for strategies capable of directly and permanently correcting the underlying genetic defect. Recent advances in genome editing have positioned CRISPR-based technologies as promising candidates for this objective. Rather than functioning as a single approach, gene-editing platforms encompass a spectrum of strategies—including exon deletion, exon reframing, base editing, and prime editing—each with distinct advantages depending on the mutational context. In particular, the emergence of precision editing tools has enabled controlled nucleotide-level modifications, expanding the range of correctable mutations while reducing reliance on double-strand DNA breaks. In this review, we adopt a comparative and translational perspective to evaluate gene-editing strategies for DMD. We examine how different approaches align with specific mutation types, summarize key findings from preclinical studies, and analyze the major barriers to clinical implementation, including delivery efficiency, immune responses, editing durability, and genomic safety. We further discuss emerging innovations in editing technologies and delivery systems that aim to address these limitations. Collectively, this work reframes gene editing as a decision-oriented and application-driven therapeutic framework. Continued integration of advances in genome engineering, delivery platforms, and muscle biology will be essential to translate these technologies into safe, effective, and durable treatments capable of altering the clinical trajectory of DMD.

## 1. Introduction

Duchenne muscular dystrophy (DMD) (OMIM: #310200) represents one of the most formidable challenges in genetic medicine, not only because of its severe and progressive nature but also due to the difficulty of achieving durable therapeutic correction in postmitotic tissues such as skeletal and cardiac muscle [1,2]. Despite decades of research and the development of multiple therapeutic approaches, a fundamental limitation remains: most interventions fail to provide sustained, body-wide restoration of dystrophin at levels sufficient for long-term functional benefit. This gap underscores a critical disconnect between molecular correction and clinical efficacy in DMD [3,4].

A major obstacle arises from the genetic and biological complexity of the disease. The DMD gene is highly susceptible to a wide spectrum of mutations—including deletions, duplications, and point mutations—distributed across its large genomic locus, necessitating either mutation-specific or broadly adaptable therapeutic strategies [1,5,6]. In parallel, the unique characteristics of muscle tissue—including its large mass, limited regenerative capacity, and the requirement for efficient systemic delivery—pose significant barriers to effective treatment. Consequently, even strategies that achieve measurable correction at the DNA or RNA level often fail to translate into robust and widespread functional recovery [7,8].

Current therapeutic approaches illustrate these challenges. Modalities such as exon skipping, gene replacement, and pharmacological interventions have demonstrated the feasibility of restoring dystrophin expression; however, their clinical benefits remain constrained by factors such as incomplete protein restoration, transient therapeutic effects, and inefficient delivery to target tissues [9,10,11]. These limitations have driven a shift in focus toward genome-targeted interventions capable of directly addressing the underlying genetic defect with the aim of achieving stable and potentially lifelong therapeutic outcomes.

Among these, gene-editing technologies have emerged as promising candidates by enabling precise modification of disease-causing mutations at their genomic source [12,13]. However, not all editing strategies offer equivalent advantages. Approaches relying on double-strand DNA breaks, such as CRISPR/Cas9-mediated editing, can restore the reading frame but are associated with variability in repair outcomes and potential safety concerns, including off-target effects and unintended insertions or deletions [14,15]. In contrast, next-generation precision editing platforms—including base editing and prime editing—have been developed to enable more controlled and predictable sequence modifications without introducing double-strand breaks, thereby potentially improving safety and versatility [15,16].

This evolving technological landscape raises a critical question: which gene-editing strategies are best suited to address the diverse mutational spectrum of DMD, and what factors will ultimately determine their translational success? Addressing this question requires moving beyond descriptive summaries of individual technologies toward a more integrative and application-driven evaluation framework.

In this review, we adopt a perspective that emphasizes the comparative and translational relevance of gene-editing approaches in DMD. We examine how different strategies align with specific mutational contexts, highlight recent advances in precision genome editing, and discuss key barriers—including delivery, immune responses, and long-term safety—that must be overcome to enable clinical implementation. By framing gene editing within a decision-oriented and translational context, this work aims to provide a clearer roadmap for the development of next-generation therapeutic strategies for DMD.

## 2. Genetic Basis and Pathophysiological Mechanisms of DMD

Duchenne muscular dystrophy (DMD) is an X-linked recessive dystrophinopathy caused by the absence of dystrophin, a protein (Uniprot P11532) encoded by the *DMD* gene located at Xp21.2-p21.1 [17]. Dystrophin is a rod-shaped cytoskeletal protein situated on the intracellular surface of the sarcolemma, where it functions as an organizational hub for the dystrophin-associated protein complex (DAPC) [18]. From a genetic perspective, the *DMD* gene contains four internal promoters, including Dp260, Dp140, Dp116, and Dp71, and produces approximately 11.4-kilobase full-length cDNA and the 427 kDa full-length dystrophin protein. Each promoter is responsible for producing non-muscle dystrophin isoforms that lack the N-terminal domain. Moreover, an additional isoform is generated via the Dp40 promoter, which initiates alternative splicing and polyadenylation at the 3′ end of the gene. The dystrophin protein consists of four primary functional domains: (i) an N-terminal region with homology to the actin-binding domains of α-actinin, encoded by exons 1 to 8 (ABD); (ii) a central rod domain comprising 24 spectrin-like repeats interspersed with four hinge regions, encoded by exons 8 to 64; (iii) a cysteine-rich domain encoded by exons 64 to 70 (CR); and (iv) a C-terminal domain encoded by exons 71 to 79 [19]. Specifically, dystrophin interacts with the transmembrane protein β-dystroglycan through its C-terminal cysteine-rich domain. β-dystroglycan, in turn, binds to α-dystroglycan, which is exposed on the extracellular surface of the sarcolemma and connects with laminin complexes in the extracellular matrix. On the intracellular side, dystrophin associates with filamentous γ-actin, intermediate filaments, and the microtubule network in the sarcoplasm via its N-terminal domain. These connections enable dystrophin to establish a mechanical link between the extracellular matrix and the actin cytoskeleton [20,21,22,23]. The primary role of dystrophin is to function as a “shock absorber,” safeguarding muscle fibers from contractile-induced damage [24]. Additionally, dystrophin contributes to various signaling pathways through its interactions within the DAPC. These include nitric oxide signaling via neuronal nitric oxide synthase activity, the MAP kinase pathway [25], and MARK2 kinase signaling, which plays a role in regulating the polarity of muscle satellite cells [26].

The loss of dystrophin disrupts the dystrophin-associated protein complex (DAPC), causing many of its components to mislocalize from the sarcolemma [18] and become downregulated [27]. For instance, sarcoglycans are both mislocalized and expressed at reduced levels in dystrophin-deficient muscle [27,28]. Disruption of sarcoglycans is linked to various forms of limb-girdle muscular dystrophy, highlighting the critical role of DAPC integrity in preventing muscle pathology [29]. Dystrophin deficiency also results in increased calcium influx, oxidative stress, and myonecrosis. Dystrophic muscle is characterized by cycles of degeneration and regeneration, accompanied by persistent inflammation. In the early stages of disease, myofiber loss is temporarily balanced by compensatory regeneration driven primarily by satellite cells. However, as the disease progresses, muscle quality declines due to extensive fibrosis and adipose tissue deposition, which gradually replace myofibers. This creates a non-productive environment that impairs satellite cell-mediated regeneration, leading to functional exhaustion. Notably, the number of satellite cells and their regenerative potential are significantly reduced in dystrophic muscle [30,31,32].

**Figure 1:**
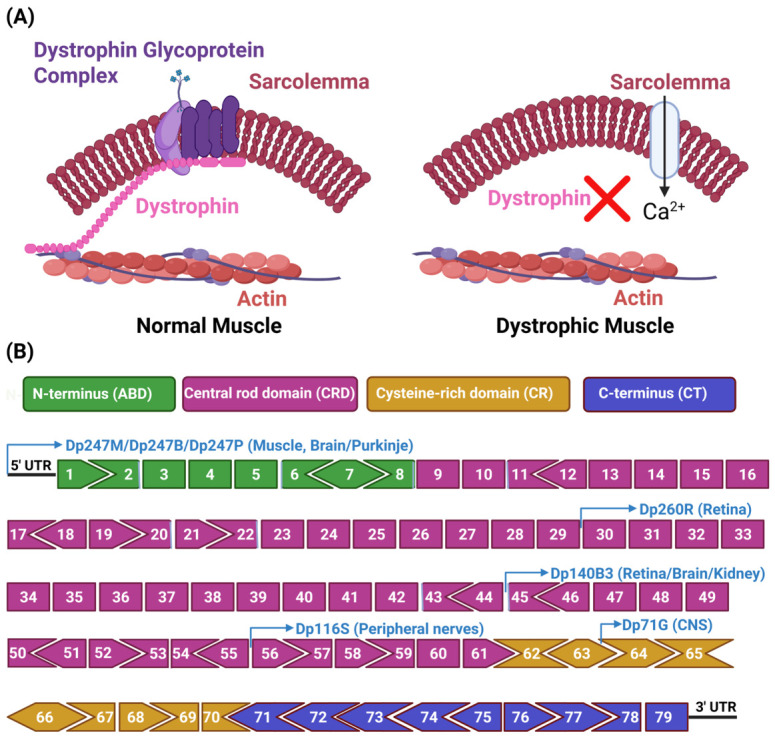
Structure of the dystrophin complex and *DMD* gene. (**A**) Schematic representation of the dystrophin-associated protein complex in both normal and DMD muscles. In normal muscle, dystrophin is localized beneath the sarcolemma, where it interacts with associated proteins to form a stable complex. In DMD, the absence of dystrophin disrupts this complex, leading to compromised membrane integrity and progressive muscle fiber damage during contraction. (**B**) Diagrammatic overview of the *DMD* gene structure. Each exon is color-coded according to the protein domain it encodes: the actin-binding domain (ABD), hinge regions, central rod domain, cysteine-rich (CR) region, and the C-terminal (CT) domain. The shapes of the exons illustrate the distribution of codons across exon boundaries, ensuring proper alignment for an in-frame mature *DMD* transcript. Arrows denote the positions of transcription start sites corresponding to different dystrophin isoforms.

The *DMD* gene, one of the largest known human genes (~2.2 Mb), has a high rate of de novo mutations. Common pathogenic mutations include whole exon deletions (~68%), exon duplications (~11%), and nonsense mutations (~10%) [33,34,35]. While mutations can occur throughout the gene, two mutation hotspots are located between exons 3 and 19 and exons 45 and 55 [36,37]. The *DMD* gene consists of 79 exons, many of which encode the 24 spectrin-like repeat domains within dystrophin’s central rod region. These domains exhibit functional redundancy, meaning that in many cases, certain deletions do not completely abrogate dystrophin function. The structural organization of the dystrophin protein and the DMD gene is illustrated in Figure 1.

Crucially, in-frame exon deletions that preserve the translation reading frame result in an internally deleted but partially functional dystrophin protein. This outcome is associated with Becker muscular dystrophy (BMD) [38,39], a milder dystrophinopathy compared to Duchenne muscular dystrophy (DMD). Patients with BMD typically have later disease onset, milder pathology, and longer life expectancy, although dilated cardiomyopathy often develops in the fourth decade of life [40]. In some younger patients, dilated cardiomyopathy may be the initial presentation of BMD [41]. Patients with BMD produce dystrophin at reduced levels or generate partially functional dystrophin due to varying degrees of internal in-frame deletions [42]. In rare cases, individuals with large internal deletions can remain effectively weakly symptomatic [38]. These findings have driven the development of therapeutic strategies aimed at restoring dystrophin expression to convert the severe DMD phenotype into the milder BMD phenotype [43].

## 3. Approved Therapies for DMD

The current clinical practice for Duchenne muscular dystrophy (DMD) patients relies on a multidisciplinary approach, incorporating corticosteroids [44,45], truncated dystrophin (mini and micro) [7,46,47,48], exon skipping therapies [49,50,51,52], gene therapy [7,53,54], utrophin modulators [55,56], myostatin inhibitors [57,58,59,60] and supportive therapy [61,62,63,64] such as physical therapy, respiratory therapy and cardiomyopathy management [65,66,67]. Corticosteroid treatments are commonly used to alleviate secondary symptoms of DMD, such as inflammation, impaired angiogenesis, fibrosis, disrupted calcium homeostasis, and mitochondrial dysfunction [68,69]. Corticosteroids, such as prednisone [45] and deflazacort [70], have been shown to improve muscle strength and preserve functional abilities. However, these treatments are not curative [44,45,70]. However, despite providing minimal improvement to the DMD phenotype, long-term corticosteroid use is associated with significant adverse effects. Additionally, the optimal dosing strategies to maximize therapeutic efficacy while minimizing side effects remain uncertain. These limitations have driven interest in molecular therapies that address the underlying genetic cause of DMD.

A promising therapeutic strategy for DMD involves the expression of a semi-functional dystrophin protein to mimic a BMD-like phenotype. Truncated dystrophin proteins, which lack exons encoding portions of the central rod domains, are designed to fit within the limited packaging capacity of adeno-associated viruses (AAVs) for systemic delivery. This innovation has led to the development of various mini- and micro-dystrophin constructs for therapeutic applications [7,46,47,48]. Currently, these truncated dystrophins are being evaluated in several clinical trials [71,72,73,74,75]. Notably, AAV-mediated micro-dystrophin gene therapy has now reached clinical translation, with delandistrogene moxeparvovec (Elevidys) receiving accelerated approval from the U.S. Food and Drug Administration (FDA) in 2023, followed by expanded approval in 2024 for the treatment of DMD [76].

Patients with BMD, who harbor in-frame mutations of the *DMD* gene, typically experience milder symptoms. This has inspired the development of exon skipping therapies, which aim to restore the open reading frame (ORF) and alleviate some symptoms in DMD patients [42,77,78,79]. Exon skipping is achieved using antisense oligonucleotides (AONs) [80,81,82]—short, synthetic, single-stranded nucleic acid analogs designed to bind to mRNA at specific sites and modulate splicing. Currently, there are four FDA-approved AON therapies for DMD [83,84]: eteplirsen (exon 51 skipping) [49], golodirsen (exon 53 skipping) [50], viltolarsen (exon 53 skipping) [51], and casimersen (exon 45 skipping) [52].

Despite their promises, AON therapies have significant drawbacks. Approved ASOs are based on phosphorodiamidate morpholino oligomer (PMO) chemistry, which is uncharged and does not bind to serum proteins. Consequently, these ASOs exhibit poor bioavailability and are rapidly cleared by the kidneys following intravenous administration [85]. Their effects are temporary, requiring repeated administration, which leads to substantial treatment costs. Furthermore, AONs often exhibit poor delivery and limited uptake into critical tissues, such as skeletal muscle and heart [86]. Only a small fraction of the administered dose reaches dystrophic skeletal muscle, resulting in low levels of exon skipping and minimal restoration of dystrophin, even at high intravenous doses, e.g., weekly doses of 30 mg/kg for eteplirsen, casimersen, and golodirsen, as well as 80 mg/kg for viltolarsen. Although dystrophin levels in the heart have not been measured in patients, findings from animal model studies suggest that they are negligible at the currently approved dosing levels [85,87,88,89]. These therapies are also mutation-specific, making them unsuitable for many DMD patients. Collectively, these challenges constrain the effectiveness and accessibility of AON-based treatments [90].

However, all these approaches have demonstrated only limited clinical benefits and fail to address the underlying genetic cause of the disease, preventing the lifelong restoration of dystrophin expression. In contrast, gene-editing therapy offers the potential for a one-time, permanent correction of the DMD gene, eliminating the need for ongoing treatments. By addressing many of the limitations associated with AONs, gene-editing technology represents a groundbreaking therapeutic strategy with the potential to overcome existing barriers and revolutionize DMD treatment. An overview of FDA-approved therapies for DMD is provided in Table 1.

## 4. Gene Editing Therapeutic Strategy

**Figure 2:**
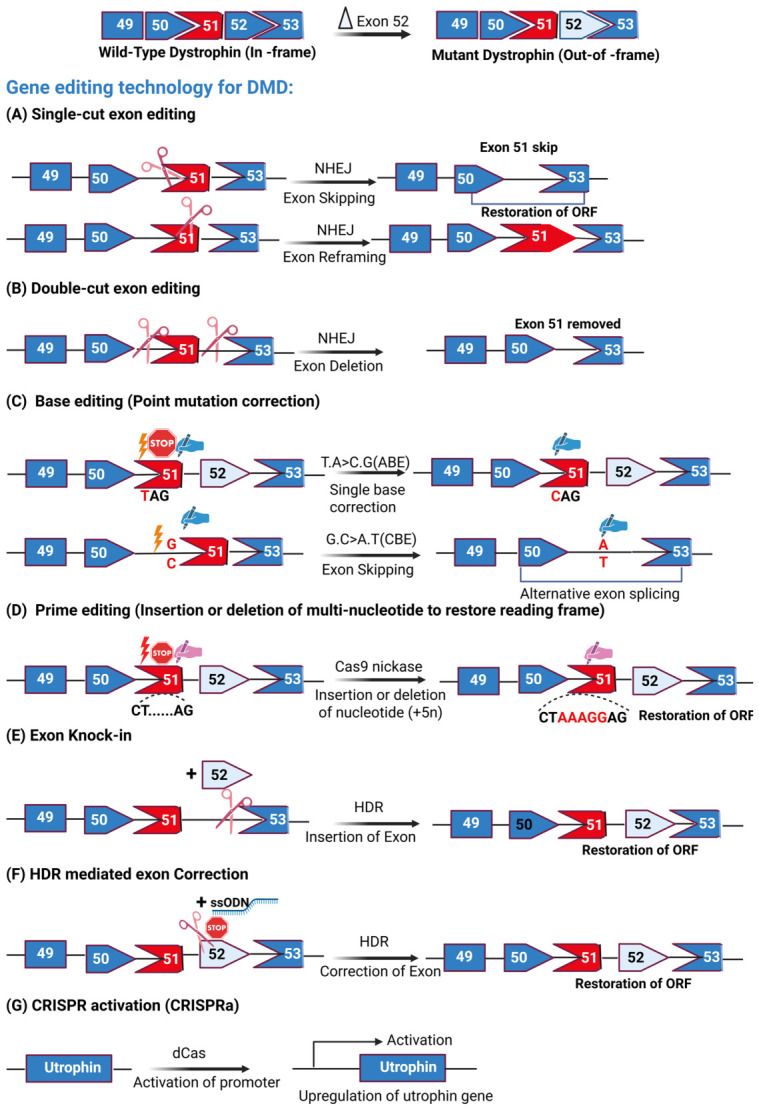
Overview of gene editing therapeutic approaches for Duchenne muscular dystrophy (DMD). These strategies include non-homologous end joining (NHEJ)-mediated editing, such as single-cut exon skipping, double-cut exon deletion, and exon reframing, along with homology-directed repair (HDR)-based exon knock-in and precise exon correction. In addition, nucleotide-level modifications are achieved through base editing and prime editing technologies. The schematic also highlights CRISPR/dCas-mediated transcriptional activation of utrophin as an alternative therapeutic approach. Panels illustrate single-cut exon editing (**A**), double-cut exon editing (**B**), nucleotide editing via base and prime editing (**C**,**D**), exon knock-in (**E**), HDR-mediated correction (**F**), and CRISPR activation (**G**).

Gene editing technologies have revolutionized the field of molecular medicine by enabling precise modifications to the genome, offering new therapeutic opportunities for DMD. These technologies include Zinc Finger Nucleases (ZFNs) [119,120,121] and transcription activator-like effector nucleases (TALENs) [121,122], CRISPR/Cas9 systems [121,122,123], base editing [124,125,126,127,128], and prime editing [128,129,130,131], which provide tools to target and repair DMD-causing mutations. An overview of these gene-editing approaches is presented in Figure 2.

### 4.1. CRISPR/Cas9 System

Rather than functioning as a single uniform platform, CRISPR-based systems now represent a diverse and adaptable toolkit for genome engineering, with distinct variants offering different advantages for therapeutic applications in Duchenne muscular dystrophy (DMD). The clinical potential of these systems lies not only in their ability to induce targeted genomic modifications but also in the flexibility with which they can be tailored to specific genetic contexts. Originally derived from a bacterial adaptive immune mechanism against viral invasion [132], CRISPR technology has been repurposed into a programmable genome-editing platform capable of introducing site-specific DNA modifications [133,134]. At its core, the CRISPR/Cas9 system relies on the coordinated action of a sgRNA and a Cas endonuclease to recognize genomic targets and induce double-stranded DNA breaks (DSBs) in the presence of a protospacer-adjacent motif (PAM) sequence [135].

Various Cas endonucleases, derived from different bacterial species, are utilized in CRISPR systems, each with a unique PAM sequence. Different Cas orthologs provide varying degrees of targeting flexibility, efficiency, and compatibility with delivery systems. The widely used SpCas9 enzyme recognizes short PAM sequences such as 5′-NGG-3′, enabling broad genomic accessibility [133,134,136]. In contrast, smaller Cas proteins such as SaCas9 offer advantages in vector-based delivery due to reduced size, albeit with more restrictive PAM requirements [137,138,139]. Even more compact variants, such as CjCas9, further expand delivery feasibility but impose additional constraints on target site selection due to longer and more complex PAM sequences [140].

These trade-offs highlight the importance of selecting appropriate Cas variants based on both genomic and translational considerations [141,142]. Structure-guided and directed evolution approaches have further expanded the targeting scope of CRISPR systems by modifying PAM compatibility and enhancing DNA recognition fidelity [143,144,145,146]. These developments are particularly relevant for therapeutic applications, where minimizing unintended genomic alterations is critical. Beyond nuclease activity, functional diversification of CRISPR systems has enabled alternative editing strategies that move away from conventional DSB-dependent mechanisms. Catalytically modified forms of Cas9, including nickase (nCas9) and nuclease-dead Cas9 (dCas9), reduce or eliminate double-strand cleavage, thereby improving safety profiles [147,148]. When fused with additional functional domains, these variants enable new classes of genome manipulation, including base editing and transcriptional modulation [149,150]. Such approaches provide greater control over editing outcomes and represent a shift toward precision genome engineering strategies that may be better suited for clinical translation.

### 4.2. Base Editing

Base editing (BE) offers a promising approach that enables precise and permanent genome modifications without the need to create DNA DSBs [151,152], thus overcoming the limitations of the CRISPR/Cas system [153]. Instead of employing a Cas9 nuclease, the base editing (BE) technique uses Cas9 nickase, which specifically cleaves a single DNA strand [154]. This system incorporates specialized enzymes, such as cytidine deaminase and deoxyadenosine deaminase, to modify specific nucleobases [155]. Cytidine deaminase catalyzes the deamination of cytosine to uracil, a process protected by the addition of uracil glycosylase inhibitor (UGI), which prevents uracil from being removed by uracil-DNA glycosylase. As the Cas9 nickase creates a bubble by unwinding the target DNA, cytidine deaminase acts on the exposed cytosines within the unpaired DNA, typically within a five-base window. This results in the conversion of cytosine to uracil, generating a U:G mismatch [156]. To direct the cell repair machinery to replace guanine (G) on the opposite strand with adenine (A), the Cas9 nickase introduces a nick on the G-containing strand. This encourages the cell to use the uracil-containing strand as a template, ultimately converting the C:G base pair into a U:A (or T:A) base pair if this modification proceeds correctly. Similarly, the adenine base editor (ABE) functions like the cytosine base editor (CBE); however, it uses instead deoxyadenosine deaminase. This enzyme catalyzes the deamination of adenine to inosine (I), which is interpreted as guanine (G) by the DNA repair system. Following a similar repair pathway as CBE, the A:T base pair is effectively converted into a G:C base pair [157]. Despite the low risk of producing indels and its high efficiency in nucleotide substitution, base editing (BE) still carries the potential risk of unintended edits. These risks stem from limitations in targeting due to stringent PAM requirements, bystander editing within the activity window, and possible off-target effects [158].

### 4.3. Prime Editing

Prime editing (PE) is another nucleotide editing technology that allows precise and permanent genome modifications without the need for DNA double strand cleavage [16]. A key distinction between the standard CRISPR/Cas9 system and prime editing (PE) lies in the inclusion of reverse transcriptase (RT), an essential component of the PE system. The PE system comprises three key elements: a fusion protein of Cas9 nickase and an engineered RT, a prime editing guide RNA (pegRNA), and a nicking guide RNA (ngRNA). The pegRNA directs the fusion protein to the target DNA strand, which is then nicked by the Cas9 nickase. Upon hybridization with the nicked strand using its primer binding site (PBS), the reverse transcriptase template (RTT) sequence of the pegRNA serves as a template for reverse transcription, resulting in the formation of a new DNA flap critical for precise genetic modifications. This process involves the incorporation of a newly synthesized 3′ strand flap while the original 5′ strand is degraded [159,160]. To further improve the efficiency of genetic alterations, the ngRNA guides the Cas9 nickase part of the Cas9-RT fusion protein to nick the strand opposite the flap-containing strand. This step enhances the likelihood of repairing the non-edited strand, ultimately producing a fully edited homoduplex double-stranded DNA [149,161].

PE was developed to address the limitations of base editors (BEs) and the CRISPR/Cas9 system. It enables genome modification to correct nearly any undesired change, including all 12 types of transition and transversion point mutations, as well as insertions and deletions (indels), without the need for double-strand breaks (DSBs) or donor DNA templates. PE is reported to have the potential to correct up to 89% of over 75,000 disease-associated variants in humans [162]. However, despite its advantages, such as performing genome editing without DSBs or donor DNA, several challenges remain, including the need for PAMs located near the nucleotides to be modified.

## 5. Gene Editing Therapeutic for DMD

### 5.1. Gene Editing via CRISPR/Cas9

CRISPR/Cas9 gene editing holds the potential to permanently correct mutations responsible for DMD and significantly alleviate the disease’s pathology. CRISPR/Cas9-mediated double-strand breaks (DSBs) can be repaired by two primary endogenous pathways: non-homologous end joining (NHEJ) and homology-directed repair (HDR) [163]. HDR relies on an exogenous DNA template to achieve precise editing at the target site. In the context of Duchenne muscular dystrophy (DMD), HDR can facilitate the replacement of mutated exon(s) through an exon knock-in approach, potentially restoring the full-length dystrophin protein [37].

While HDR’s precision is advantageous, it has significant limitations in postmitotic cells, such as skeletal muscle cells, where it occurs at very low frequencies [12,64]. Furthermore, the size of the DNA template that can be delivered is constrained, making it challenging to address large deletions spanning multiple exons. As a result, HDR is generally not the preferred approach for DMD therapeutics. In postmitotic cells, the predominant DSB repair pathway is NHEJ [64]. Although considered less accurate than HDR, NHEJ introduces small insertions or deletions (indels) at the DSB site. These indels can restore dystrophin expression through various mechanisms, including: (i) Exon skipping (single-cut): a single guide RNA (sgRNA) targeting an exon–intron junction induces the skipping of the out-of-frame exon. (ii) Exon deletion (double-cut): two sgRNAs flanking the exon(s) to be removed can excise the mutated region, restoring the open reading frame (ORF). (iii) Exon reframing (single-cut): a single cut introduces indels that reframe the transcript to produce functional dystrophin (Figure 2) [12,64,65,66]. These approaches highlight the versatility of CRISPR/Cas9 for addressing DMD mutations through NHEJ-mediated repair, offering practical strategies despite the pathway’s inherent imprecision.

### 5.2. In Vivo and In Vitro Gene Editing for DMD

The first proof-of-concept for in vivo CRISPR-mediated gene editing in Duchenne muscular dystrophy (DMD) was demonstrated by Long et al. in 2014 [67,164]. Utilizing the well-established mdx mouse model, which carries a nonsense mutation in exon 23 of the *dmd* gene [68], the researchers microinjected zygotes with SpCas9, a single guide RNA (sgRNA), and an exogenous DNA template to facilitate homology-directed repair (HDR) [67]. The resulting offspring exhibited mosaicism in dystrophin expression, with correction rates ranging from 2% to 100%. Although germline gene editing is not ethically permissible in humans, this foundational study shifted the research focus toward postnatal gene editing strategies. A key insight from Long et al.’s work is that complete correction is not necessary to achieve therapeutic benefit; they observed that approximately 15% gene editing was sufficient to restore functional levels of wild-type (WT) dystrophin protein. This outcome arises from the syncytial architecture of skeletal muscle, which enables corrected nuclei to disseminate dystrophin protein across the entire muscle fiber, thereby delivering significant therapeutic benefits even when only a subset of nuclei is corrected.

### 5.3. Single-Cut Exon Editing

#### 5.3.1. Exon Skipping Strategies

The single-cut exon skipping strategies for Duchenne muscular dystrophy (DMD) are mutation-dependent and applicable to specific patient subsets [165]. The single-cut exon skipping approach employs a single guide RNA (sgRNA) and CRISPR/Cas9 to introduce a single double-strand DNA break (DSB) at intron–exon junctions or splice signal sequences of the *DMD* gene [166]. Subsequent repair by the endogenous non-homologous end joining (NHEJ) pathway generates small insertions or deletions (INDELs) that disrupt splice donor or acceptor sites, resulting in the skipping of the targeted exon during mRNA processing. This restores the open reading frame (ORF) and enables the production of a truncated yet functional dystrophin protein [167].

Compared with double-cut editing, the single-cut method is simpler and safer, as it requires only one sgRNA and induces fewer genomic alterations. The PAM sequence of SpCas9 is compatible with universal splice acceptor sites, facilitating efficient targeting [37]. According to the Leiden DMD mutation database, approximately 70–80% of DMD patients could benefit from exon skipping therapy [168,169], highlighting its strong therapeutic potential as demonstrated in multiple in vitro and in vivo studies.

Li et al. provided the first demonstration of single-cut exon editing in an in vitro model of Duchenne muscular dystrophy (DMD). In their study, patient-derived induced pluripotent stem cells (iPSCs) carrying a deletion of exon 44 in the *Dmd* gene were utilized [122]. This deletion caused a frameshift mutation in the open reading frame (ORF), resulting in the loss of functional dystrophin protein. To correct this defect, the researchers employed single guide RNAs (sgRNAs) targeting sequences flanking exon 44, either to disrupt the splice acceptor site of exon 45, thereby promoting its skipping and reconnecting exons 43 and 46, or to induce small indels that realign the reading frame. Following correction, the edited the induced pluripotent stem cells (iPSCs) were differentiated into skeletal muscle cells that successfully expressed full-length dystrophin, confirming functional gene restoration [122].

In another in vitro study, Long et al. [170] employed patient-derived iPSCs harboring deletions of exons 48–50, which create an out-of-frame exon 51 containing a premature stop codon [171]. Using sgRNAs directed to the splice acceptor site of exon 51, the team induced exon skipping, effectively restoring the dystrophin reading frame. The corrected iPSCs were subsequently differentiated into cardiomyocytes, which exhibited restored dystrophin expression. Moreover, three-dimensional engineered heart muscle (EHM) generated from these corrected cells showed not only dystrophin restoration but also enhanced contractile strength. Remarkably, correction of only 30–50% of cardiomyocytes was sufficient to rescue the EHM phenotype to near-normal functional levels [171].

Several studies have confirmed the high efficiency of single-cut sgRNA strategies in restoring dystrophin expression in in vivo models. For example, Amoasii et al. successfully treated ΔEx50 mice [172] and dogs [173] using a single-cut exon skipping approach targeting exon 51. This method reframed the dystrophin transcript by inducing exon 51 skipping, restoring up to 90% of dystrophin protein in skeletal and cardiac muscles. The treatment substantially improved muscle strength and reduced dystrophic pathology, with no evidence of immune responses, off-target editing, or hepatotoxicity. Similarly, Min et al. (2019) [174] achieved approximately 90% dystrophin restoration across multiple muscle groups, including the heart, within four weeks following a single systemic administration of AAV9-encoded gene-editing components in ΔEx44 mice [175].

More recently, Rok et al. (2024) [176] reported that targeting the splice donor site of exon 55 with one sgRNA enhanced editing efficiency and dystrophin recovery compared to dual-guide exon excision strategies [170]. Although this approach effectively prevented early-onset cardiac dysfunction in Δ52–54 DMD mice, dystrophin restoration in skeletal muscles was insufficient to improve motor performance, emphasizing the need for optimized AAV delivery to peripheral tissues. Furthermore, the therapeutic efficacy of single-cut editing can vary by mutation type. For instance, targeting the splice donor site of exon 44 using SpCas9 restored dystrophin in approximately 60% of myofibers in DMD ΔEx45 mice, but only ~36% in DMD ΔEx43 mice [177]. These findings highlight the necessity of refining sgRNA design and delivery systems to fully realize the therapeutic potential of single-cut exon editing in DMD.

#### 5.3.2. Exon Reframing Strategy

Exon reframing is a single-cut CRISPR/Cas9-based genome-editing strategy designed to restore the open reading frame (ORF) in genes disrupted by frameshift mutations through the introduction of small insertions or deletions (indels) generated by the non-homologous end joining (NHEJ) repair pathway. By using a single guide RNA (sgRNA) to induce a targeted double-strand break, exon reframing exploits the fact that approximately one-third of NHEJ-mediated indels can realign the reading frame, enabling production of a larger and more functional protein compared with exon skipping approaches [166,167]. The predictability of NHEJ outcomes, partially influenced by sequence context, including the fourth nucleotide upstream of the protospacer-adjacent motif (PAM), enhances the precision of this strategy, while iterative Cas9 activity can further enrich for productive reframing events when uncorrected alleles remain targetable. Optimization of sgRNA design has improved reframing efficiency, as exemplified by Min et al. [170], who engineered a strategy in which a single adenosine insertion is preferentially generated through a one-nucleotide overhang, enabling reliable ORF restoration in single-nucleotide frameshift mutations [178]. In the context of Duchenne muscular dystrophy (DMD), Zhang et al. demonstrated that single-cut CRISPR/Cas9-mediated exon reframing using a compact Cas9 system compatible with single-vector AAV delivery restored the dystrophin ORF in human iPSC-derived cardiomyocytes and DMD mouse models, resulting in expression of internally truncated but functional dystrophin and corresponding improvements in muscle performance [174,176]. The long-term durability of this approach was established by Karri et al., who reported stable genomic correction and sustained dystrophin expression for up to 18 months post-editing with minimal off-target effects, indicating persistent functional benefit in aging muscle tissue [179]. Extending these findings, Durbacz et al. demonstrated that optimized single-cut CRISPR editing in patient-derived iPSCs and a humanized DMD mouse model with exon 52 deletion restored dystrophin expression across multiple skeletal muscle groups and the heart and ameliorated key DMD pathological features and functional deficits, supporting the translational potential of single-cut exon reframing strategies [180].

### 5.4. Double-Cut Exon Deletion

A major therapeutic challenge in Duchenne muscular dystrophy (DMD) is the high prevalence of out-of-frame deletions, which disrupt the dystrophin reading frame in approximately 65–72% of patients [33]. This mutational pattern has driven the development of strategies aimed at restoring the open reading frame rather than replacing the entire gene. Among these, double-cut exon deletion has emerged as a genome-editing approach designed to convert severe DMD mutations into Becker-like, partially functional dystrophin phenotypes. Unlike mutation-specific strategies, double-cut editing offers a broadly applicable solution by removing selected exons using two guide RNAs (sgRNAs) flanking the target region. This approach enables the generation of internally truncated yet functional dystrophin proteins and can theoretically address a wide range of mutation types, including deletions, duplications, and certain point mutations [181,182]. Its mutation-independent nature makes it particularly attractive for expanding therapeutic coverage across diverse patient populations.

However, the success of this strategy depends strongly on the structural tolerance of the dystrophin protein to internal deletions. Not all exon combinations yield functional outcomes, and selecting appropriate genomic regions for excision is therefore critical. For example, the N-terminal hotspot spanning exons 3–9 has been extensively investigated due to its clinical relevance [183,184]. While large deletions in this region might be expected to destabilize dystrophin, clinical observations of patients with Δ3–9 deletions reveal relatively mild Becker muscular dystrophy (BMD)-like phenotypes, likely due to preservation of key functional domains such as the actin-binding site [34]. Experimental studies support this concept: Kyrychenko et al. demonstrated that among several multi-exon deletion strategies, Δ3–9 excision most effectively restored dystrophin function and improved calcium handling in iPSC-derived cardiomyocytes, whereas other deletions (e.g., Δ7–11) produced unstable proteins with limited functional recovery [184].

A similar principle applies to the exon 45–55 mutational hotspot, which represents one of the most promising targets for therapeutic exon excision. In-frame deletions within this region could potentially benefit more than 60% of DMD patients [185]. Early work demonstrated that excision of exon 51 or the entire 45–55 region restored dystrophin expression in patient-derived myoblasts, although editing efficiency decreased with increasing deletion size [186,187]. Early work demonstrated that excision of exon 51 or the entire 45–55 region restored dystrophin expression in patient-derived myoblasts, although editing efficiency decreased with increasing deletion size. Subsequent studies confirmed that Δ45–55 editing not only restored dystrophin expression but also improved muscle cell function and stability, accompanied by molecular signatures consistent with milder disease phenotypes [181]. More recent investigations further showed that multi-exon excision strategies can correct defects in myogenic differentiation, reinforcing their functional relevance 195. Additional deletion designs, including Δ44–54, Δ46–54, and Δ48–57, have also been explored, with certain configurations (e.g., Δ48–57) preserving structural features such as properly phased spectrin-like repeats, which are critical for dystrophin stability [188,189,190]. The translational feasibility of double-cut strategies has been demonstrated across multiple in vivo models. Genome editing using viral delivery systems has enabled sustained dystrophin restoration in both skeletal and cardiac tissues. For instance, exon deletions introduced via AAV-mediated delivery restored dystrophin expression for extended periods in mdx mouse models [189], while multi-exon excision strategies achieved long-term cardiac expression without detectable toxicity [190]. Larger animal models further support these findings, with systemic delivery of CRISPR components restoring dystrophin to therapeutically relevant levels and improving muscle pathology and survival [191]. However, editing outcomes remain tissue-dependent, with cardiac muscle often showing higher correction efficiency than skeletal muscle [192,193] and some approaches yielding limited restoration despite successful genomic excision [194].

Despite its broad applicability and demonstrated efficacy, double-cut exon deletion faces important technical and safety challenges. The requirement for simultaneous cleavage at two genomic sites introduces additional complexity, reducing overall editing efficiency compared with single-cut approaches and increasing the risk of unintended genomic alterations [195]. Moreover, large genomic deletions require precise end joining over extended distances, which may further compromise consistency of editing outcomes. The use of multiple sgRNAs also elevates the potential for off-target effects and chromosomal rearrangements, raising safety concerns that must be carefully addressed in clinical applications.

Taken together, double-cut exon deletion represents a powerful but context-dependent strategy for DMD gene correction. Its effectiveness relies on careful selection of target regions, optimization of delivery systems, and mitigation of safety risks. These considerations highlight the need for continued refinement and comparison with emerging precision editing technologies that may offer improved control over editing outcomes.

### 5.5. Base Editing and Prime Editing

The emergence of base editing and prime editing has significantly expanded the therapeutic landscape for Duchenne muscular dystrophy (DMD), particularly for patients carrying single-nucleotide variants, which account for approximately 25–35% of cases [168]. Unlike double-strand break (DSB)-dependent approaches, these precision editing technologies enable targeted nucleotide modifications with greater control over editing outcomes, making them especially attractive for mutation-specific correction strategies.

From a therapeutic perspective, base editing has demonstrated strong potential for efficient and durable dystrophin restoration across multiple experimental systems. By directly converting specific nucleotides without inducing DSBs, both cytosine and adenine base editors can correct point mutations or modulate splice sites to restore the dystrophin reading frame. Early proof-of-concept studies established the feasibility of this approach. For example, adenine base editing was successfully used to induce exon skipping in ΔEx51 models, resulting in robust dystrophin restoration in both human iPSC-derived cells and mouse muscle tissue following local delivery [128]. Similarly, correction of nonsense mutations via targeted A-to-G conversion restored dystrophin expression in a subset of myofibers, demonstrating that even partial genomic correction can yield meaningful protein recovery [124]. Subsequent studies have further highlighted the scalability and efficiency of base editing, particularly in cardiac tissue. High editing efficiencies have been achieved through splice-site targeting, leading to near-complete restoration of dystrophin and associated protein complexes in iPSC-derived cardiomyocytes. In vivo, systemic or localized delivery of base editors has resulted in substantial dystrophin recovery, with some models achieving long-term expression exceeding one year following a single treatment [196]. More recent advances have focused on improving targeting flexibility and expanding editing scope. The development of NG-PAM-compatible editors has enabled correction of previously inaccessible genomic sites, with systemic delivery achieving near-complete restoration in cardiac tissue and measurable recovery in skeletal muscle [133]. Additional strategies, including targeted mutagenesis of splice-site nucleotides and “single-swap” editing approaches, have demonstrated effective dystrophin restoration across multiple mutation types and experimental models [194,196]. Notably, recent studies in humanized DMD models report widespread dystrophin recovery across key muscle groups, with restoration levels approaching those observed in wild-type tissues [197,198].

Despite these promising outcomes, base editing remains constrained by several intrinsic limitations. Dependence on PAM sequences restricts target site accessibility, while the relatively large size of editing constructs complicates efficient delivery. In addition, concerns regarding off-target deamination and long-term genomic safety, particularly for cytosine base editors, continue to require careful evaluation in preclinical and clinical settings [97,192,193].

In contrast to base editing, prime editing offers a more versatile platform capable of introducing all types of nucleotide substitutions, as well as small insertions and deletions, without requiring donor templates or inducing DSBs. This expanded editing capability is particularly advantageous for correcting diverse mutation types that are not amenable to base editing. Initial studies demonstrated that prime editing can restore the dystrophin reading frame through precise nucleotide insertion, resulting in partial recovery of dystrophin expression and improved functional outcomes in cardiomyocytes 128. However, editing efficiency is influenced by the spatial relationship between the nick site and the desired edit, posing challenges for certain target designs [199].

Efforts to optimize prime editing have focused on improving editing efficiency and expanding design flexibility. Modifications to the reverse transcription template (RTT) have been shown to enhance editing at distal sites, significantly increasing correction efficiency in patient-derived myoblasts and leading to substantial dystrophin restoration following differentiation [199]. Additional studies have demonstrated successful correction of pathogenic point mutations in human cells, with measurable genomic modification and restoration of dystrophin expression in vitro [135]. These findings underscore the potential of prime editing as a highly precise and adaptable strategy for mutation-specific correction in DMD.

Taken together, base editing and prime editing represent complementary approaches within the broader framework of precision genome engineering. Base editing offers high efficiency and strong in vivo performance for specific mutation classes, whereas prime editing provides expanded versatility and editing scope. The continued development of these technologies—particularly improvements in delivery systems, editing efficiency, and long-term safety—will be critical for translating their promise into clinically viable therapies for DMD.

## 6. Homology-Directed Exon Replacement Strategies

A fundamentally different genome editing strategy for Duchenne muscular dystrophy (DMD) involves precise exon replacement through homology-directed repair (HDR). Unlike exon skipping or reframing approaches that generate internally truncated dystrophin proteins, HDR-based editing introduces an exogenous DNA donor template at a CRISPR/Cas9-induced break site, allowing accurate correction of the endogenous locus and potential restoration of full-length dystrophin [37]. This approach is particularly relevant for mutations affecting the N-terminal actin-binding domain or the C-terminal dystroglycan-binding domain, where deletions would disrupt critical functional regions and are therefore not therapeutically acceptable [36]. Despite its conceptual appeal, HDR is intrinsically inefficient in post-mitotic tissues such as mature skeletal muscle, because it is largely restricted to dividing cells and specific phases of the cell cycle [183]. For this reason, HDR-based editing has limited applicability for direct in vivo correction of established muscle fibers. Nevertheless, it has been successfully implemented in early embryonic systems, proliferating progenitors, and pluripotent stem cells [199,200,201].

One of the earliest demonstrations of HDR-mediated DMD correction was reported in 2014 by Long C and colleagues. In this study, a single guide RNA (sgRNA) targeting exon 23 of the murine *Dmd* gene, together with a single-stranded oligodeoxynucleotide (ssODN) repair template, was microinjected into zygotes derived from mdx mice [202]. HDR-driven repair corrected approximately 41% of mutant alleles, resulting in detectable dystrophin restoration across multiple muscle groups in young mice. However, when compared directly, non-homologous end joining (NHEJ)-mediated reframing achieved even higher editing efficiencies, underscoring the relative limitations of HDR in this setting.

In a related embryonic editing approach, CRISPR components based on LbCpf1 (Cas12a), combined with a 180-base ssODN donor, were introduced into mdx zygotes to repair the same exon 23 mutation [203]. Reported correction efficiencies varied widely (8–50%), but successful editing restored full-length dystrophin in skeletal muscle, cardiac tissue, and even the brain. Importantly, corrected animals exhibited no detectable fibrosis or inflammatory pathology, indicating functional rescue at the tissue level.

Beyond point mutation repair in animal models, HDR has also been used to reconstruct missing exons in patient-derived cells. For example, CRISPR/Cas9 coupled with donor DNA templates enabled reinsertion of exon 44 into ΔEx44 human induced pluripotent stem cells (iPSCs), restoring expression of full-length dystrophin in differentiated derivatives [122]. Such stem cell-based correction strategies are particularly valuable for mechanistic studies and may hold promise for future autologous cell therapies. The limitations of HDR in differentiated muscle were further illustrated by Bengtsson NE in 2017 using the mdx4Cv model [204]. In that study, AAV6 vectors encoding SpCas9, sgRNA, and a donor template were delivered intramuscularly to target exon 53. HDR-mediated repair occurred in only 0.18% of total genomes—substantially lower than parallel NHEJ-based editing at the same locus. These findings reinforce the central challenge of HDR dependency on cell proliferation, which restricts its efficiency in mature, post-mitotic myofibers. In 2020, Mata López S and colleagues investigated intramuscular delivery of HDR-mediated CRISPR/Cas9 components in the Golden Retriever muscular dystrophy dog (GRMD) model [164]. The intervention achieved dystrophin restoration of up to 16% of normal protein levels within treated muscles. Despite measurable protein recovery, this partial correction did not translate into meaningful histopathological improvement or enhanced muscle force production. These results highlight the translational challenges of HDR-dependent editing in large, clinically relevant models and emphasize the need for substantial optimization, particularly in delivery efficiency, editing rates, and functional rescue, before HDR-based strategies can be considered viable therapeutic options for DMD.

## 7. Homology-Independent Targeted Integration (HITI)

Although homology-directed repair (HDR) enables precise exon insertion and restoration of full-length dystrophin, its dependence on cell cycle activity severely limits its applicability in post-mitotic tissues such as mature skeletal muscle [163]. This limitation is particularly relevant in Duchenne muscular dystrophy (DMD), where durable correction of differentiated myofibers is required. Nevertheless, the conceptual advantage of exon knock-in remains compelling, especially for mutations affecting the N- or C-terminal domains of dystrophin, which are essential for protein function and cannot tolerate internal deletions or truncations [36]. To overcome the cell cycle constraints of HDR, a homology-independent strategy termed homology-independent targeted integration (HITI) was developed [205]. Unlike HDR-based approaches, HITI leverages the non-homologous end joining (NHEJ) pathway, which is active in both dividing and non-dividing cells [206]. In this system, the donor DNA is engineered with Cas9 recognition sites flanking the therapeutic insert. Following Cas9-mediated cleavage of both the genomic target locus and the donor construct, the NHEJ machinery facilitates directional integration of the donor sequence into the genome. Because this process does not rely on homologous recombination, it can function efficiently in post-mitotic tissues.

The feasibility of HITI-mediated exon replacement has been demonstrated in a DMD context using the hDMDΔ52/mdx mouse model [207]. Delivery of SaCas9 together with AAV-packaged donor templates enabled insertion of the missing human exon 52, resulting in restoration of full-length dystrophin in both skeletal and cardiac muscle. Notably, intramuscular and systemic administration achieved substantial correction, with up to 50% dystrophin-positive fibers reported in individual animals. Furthermore, the same study explored the delivery of a large “super-exon” containing the final 28 exons of the *DMD* gene, suggesting a mutation-independent strategy that could theoretically benefit more than 20% of patients with DMD [207]. Collectively, HITI represents a promising exon knock-in platform that combines the precision of targeted insertion with the broader applicability of NHEJ-based repair, offering a potential path toward full-length dystrophin restoration in post-mitotic muscle tissue.

## 8. Mutation-Independent Therapeutic Strategies in DMD

In addition to directly correcting mutations in the dystrophin gene, alternative strategies aim to mitigate disease pathology by targeting modifier pathways that function independently of the specific genetic defect. One of the most extensively studied modifiers is utrophin (UTRN), a structural and functional paralog of dystrophin that shares significant homology and can substitute for several of dystrophin’s membrane-stabilizing roles [208,209]. During embryonic development, utrophin is broadly expressed at the sarcolemma, but in mature muscle it is largely replaced by dystrophin [210,211]. Experimental evidence indicates that re-elevating utrophin levels in dystrophic muscle can compensate, at least partially, for dystrophin deficiency, with therapeutic benefits increasing proportionally to expression levels and without clear toxicity concerns [212,213,214].

Because the full-length utrophin coding sequence exceeds the packaging limits of commonly used viral vectors, strategies have shifted from gene replacement toward transcriptional activation of the endogenous *UTRN* locus. CRISPR activation (CRISPRa) platforms, employing catalytically inactive Cas9 (dCas9) fused to transcriptional activators, have demonstrated encouraging results in this context. In 2016, Wojtal D utilized a dCas9-VP160 system to stimulate the *UTRN* promoter in Δexon 45–52 DMD myoblasts, achieving a 1.7- to 6.9-fold increase in utrophin expression above baseline levels [56]. In vivo validation followed when Liao HK (2017) [215] applied a related CRISPRa approach in mdx mice, reporting a 3–4-fold elevation in utrophin expression accompanied by measurable improvements in hind-limb grip strength after two months of treatment [216]. More recently, Andrysiak E (2024) [217] extended this strategy to cardiomyocytes derived from DMD patient-specific induced pluripotent stem cells (hiPSC-CMs) using a dCas9-VP64 activation system [218]. Targeting the UTRN promoter produced nearly a fourfold increase in utrophin mRNA levels and contributed to preservation of key physiological properties, including intracellular calcium handling.

Beyond transcriptional activation, post-transcriptional regulation of utrophin has also been targeted. One approach involves CRISPR/Cas9-mediated deletion of the inhibitory microRNA target region (IMTR) within the 3′ untranslated region of *UTRN*. Sengupta K (2020) [55] demonstrated that removal of this regulatory element in DMD patient-derived hiPSCs resulted in approximately a twofold increase in utrophin protein expression, with corresponding functional improvement in differentiated myotubes [219].

Efforts to modulate dystrophin expression itself through gene activation have also progressed to clinical translation. Notably, Cure Rare Disease initiated the first CRISPR-based individualized (“n-of-1”) clinical trial for DMD (NCT05514249), targeting a patient with an exon 1 deletion [215]. This approach employed AAV9-mediated delivery of dCas9-VP64 to enhance dystrophin expression. Although the patient experienced serious adverse events, including cardiac complications and acute respiratory distress syndrome, these outcomes were subsequently attributed to the high viral vector dose rather than the CRISPR activation platform itself.

Collectively, mutation-independent strategies—particularly those focused on utrophin upregulation—offer a complementary therapeutic avenue applicable to the broader DMD population. Continued refinement of delivery systems and safety profiles will be critical to realizing their full clinical potential.

## 9. CRISPR-Engineered Animal Models of DMD

Beyond its therapeutic applications, CRISPR/Cas9 has revolutionized the generation of DMD animal models. Compared with conventional gene-targeting techniques that rely on embryonic stem cells and lengthy breeding schemes, CRISPR enables rapid, precise introduction of defined mutations directly in zygotes, often within a single generation [217,220]. The ability to design sgRNAs targeting specific exons, or even multiple loci simultaneously, has significantly accelerated the production of models that closely replicate patient mutations. These advances have greatly expanded experimental platforms for studying disease mechanisms and evaluating emerging therapies. More than 60 DMD models have been described across species, ranging from invertebrates such as Caenorhabditis elegans and Drosophila to vertebrates including zebrafish, rodents, rabbits, dogs, pigs, and non-human primates [221]. Among these, murine systems remain the most widely used due to their accessibility and genetic manipulability.

### 9.1. Mouse Models

Several CRISPR-generated mouse strains reproduce clinically relevant deletions found in patients. For example, exon 50 deletion (ΔEx50) mice, developed by Amoasii L in 2017, lack dystrophin in skeletal and cardiac muscle and exhibit dystrophic pathology [222]. A modified version incorporating a luciferase reporter permits noninvasive monitoring of dystrophin restoration [223]. Similarly, exon 44 deletion (ΔEx44) mice generated by Min YL display severe muscle degeneration and are particularly useful for testing exon skipping strategies targeting the exon 43–55 hotspot [178].

Large-scale deletions have also been modeled. Egorova TV produced a ΔEx8–34 mouse carrying one of the most extensive in vivo deletions reported, recapitulating severe disease phenotypes associated with proximal mutation clusters [224]. Additional exon 23 frameshift variants created by Koo T demonstrated the feasibility of employing compact nucleases such as CjCas9 for therapeutic development [225]. Base-editing technology has likewise been used to introduce point mutations, as shown by Kim K, who generated a premature stop codon in exon 20 to model nonsense mutations [226].

Humanized mouse models further enhance translational relevance. The hDMDdel45 strain mimics exon 45 deletion mutations common in patients and serves as a platform for testing exon 45–55 skipping strategies [227]. A Δ52–54 model developed by Wong TWY (2020) [228] exhibits progressive skeletal muscle weakness and early-onset hypertrophic cardiomyopathy, features not consistently observed in traditional mdx mice [229]. More recently, Lin J engineered a knock-in model replacing murine exons 50–51 with the human exon 50 sequence, producing severe skeletal muscle fibrosis and structural disorganization [197].

Despite their utility, murine models often display milder phenotypes than human patients, partly due to robust regenerative capacity and compensatory utrophin upregulation [183,228,230]. This has motivated the development of alternative species models.

### 9.2. Rat and Rabbit Models

CRISPR-edited rats, first reported by Nakamura K in 2014, carry deletions or insertions in *Dmd* exons 3 or 16 and show complete dystrophin loss, elevated creatine kinase levels, and progressive skeletal and cardiac pathology [231,232]. Their larger size and sustained disease progression make them advantageous for functional and pharmacological studies [232,233,234].

In rabbits, Sui T disrupted exon 51 using CRISPR/Cas9, generating animals with absent dystrophin, reduced sarcolemmal protein complexes, fibrosis, and impaired cardiac function by four months of age [235]. Although physiologically relevant and cost-effective compared to larger mammals, rabbit models have not yet been widely applied in therapeutic testing [236].

### 9.3. Porcine and Canine Models

Pigs offer strong anatomical and cardiac similarity to humans. The first CRISPR-induced DMD pig, created by Klymiuk N via exon 52 deletion, exhibited severe pathology but early mortality [237]. Subsequent exon 27 targeting in Diannan miniature pigs by Yu H also resulted in profound dystrophic features but limited survival [238]. Although early lethality complicates long-term studies, porcine models remain valuable for investigating cardiac manifestations due to their physiological resemblance to humans [236,239,240].

Naturally occurring canine models, such as the Golden Retriever muscular dystrophy dog (GRMD) and CXMDJ dogs, closely parallel human disease progression, including fibrosis and cardiomyopathy [241,242]. However, high maintenance costs and breeding constraints limit widespread use [239,240,243].

### 9.4. Non-Human Primate Models

The first CRISPR-generated DMD non-human primate was reported in 2015 by Chen Y, who introduced mutations into exons 4 and 46 of the *DMD* gene in rhesus monkeys (*Macaca mulatta*) [244]. Edited embryos produced both stillborn and live mutants, with histological evidence of early dystrophic changes observed prenatally. Follow-up analysis confirmed minimal off-target editing in coding regions [245]. Although primate models provide the closest genetic and physiological match to humans, ethical considerations, high costs, and limited phenotypic data constrain their application in preclinical development [236].

Collectively, CRISPR technology has dramatically broadened the spectrum of DMD animal models, enabling precise replication of patient-specific mutations across multiple species. Each model offers distinct advantages and limitations, and together they provide complementary systems for understanding disease biology and evaluating emerging therapeutic strategies.

## 10. Challenges and Future Perspectives in CRISPR-Based Therapy for DMDRB

The recent clinical approval of a CRISPR/Cas9-based therapy for sickle cell disease represents a major milestone for genome editing and has intensified expectations for similar breakthroughs in Duchenne muscular dystrophy (DMD) [246]. However, translating genome-editing technologies into effective therapies for DMD requires overcoming a set of interconnected biological and technological challenges. These challenges are not independent but instead converge on a central goal: achieving safe, efficient, and durable restoration of dystrophin across all affected muscle tissues.

A key determinant of therapeutic success is the ability to achieve sufficient and long-lasting dystrophin expression. Notably, complete correction of all myonuclei is not required; restoration of dystrophin to as little as 4–50% of normal levels can substantially ameliorate disease severity by shifting the phenotype toward a Becker-like state [247]. However, the dynamic nature of muscle degeneration and regeneration in DMD introduces an additional layer of complexity, as newly formed fibers may dilute previously corrected nuclei over time [248,249]. This highlights the importance of targeting satellite cells, which serve as the regenerative reservoir of skeletal muscle. Encouragingly, genome editing of satellite cells in preclinical models has demonstrated sustained dystrophin expression for extended periods, suggesting a pathway toward durable therapeutic benefit [250,251,252].

At the same time, immune compatibility remains a major barrier to systemic genome editing. Current delivery strategies rely heavily on adeno-associated virus (AAV) vectors, which, despite their favorable muscle tropism, are associated with both pre-existing immunity and dose-dependent toxicity [253,254,255]. High systemic doses required for whole-body delivery can lead to serious adverse effects, including liver and kidney toxicity and cardiopulmonary complications. Efforts to mitigate these risks include the development of muscle-specific AAV capsids, transient immunosuppression protocols, and improved vector designs that enhance efficiency at lower doses [256,257,258]. In parallel, immune responses directed against Cas proteins represent an additional concern, as pre-existing immunity to commonly used nucleases have been documented in human populations [259]. Strategies such as engineering less immunogenic Cas variants and restricting expression to target tissues aim to reduce these risks [260,261], while the relatively low immunogenicity of restored dystrophin may further support therapeutic tolerance [262].

Beyond immunological considerations, genomic safety remains a critical requirement for clinical translation. Off-target editing, although less prevalent in post-mitotic tissues, continues to pose a potential risk, particularly in proliferative cell populations [263,264]. Advances in high-fidelity nucleases and refined sgRNA design have significantly improved targeting specificity [183,265,266]. Complementary computational and experimental tools now enable comprehensive genome-wide profiling of off-target effects, strengthening the safety assessment framework for therapeutic applications [168].

Efficient delivery represents another central bottleneck that intersects with all aspects of genome-editing performance. While AAV vectors remain the leading platform for in vivo delivery, their limited packaging capacity imposes constraints on the size of editing components, often necessitating the use of smaller Cas variants or dual-vector systems [255,267,268]. Alternative viral systems provide increased cargo capacity but introduce additional safety concerns, including insertional mutagenesis [269,270]. Consequently, non-viral delivery approaches are gaining increasing attention. Lipid nanoparticles (LNPs), already validated in clinical settings, have demonstrated the ability to deliver genome-editing components and restore dystrophin expression in preclinical models [271,272,273]. Emerging platforms such as gold nanoparticles and virus-like particles further expand the delivery landscape by enabling transient, non-integrating delivery of editing complexes [274].

Taken together, these challenges highlight that the future of genome editing in DMD will depend on the integration of multiple advances rather than the optimization of a single component. Emerging technologies—including programmable integration systems, twin prime editing, and CRISPR-associated transposases—offer new opportunities to enhance editing precision and durability [275,276]. In parallel, combinatorial strategies that couple genome editing with approaches such as utrophin upregulation or micro-dystrophin delivery may provide synergistic benefits and improve long-term muscle stability [218,277].

In this evolving landscape, the path toward clinical translation is increasingly defined by the ability to balance efficiency, safety, and scalability. Continued interdisciplinary innovation across genome engineering, delivery technologies, and muscle biology will be essential to determine whether genome editing can ultimately fulfill its promise as a transformative and durable therapy for DMD.

## 11. Concluding Remarks

Genome editing has redefined the therapeutic landscape of Duchenne muscular dystrophy (DMD), shifting the focus from symptomatic management toward the possibility of durable genetic correction. CRISPR/Cas9-based strategies offer a distinct advantage by directly targeting the underlying cause of the disease, in contrast to currently approved therapies such as exon skipping and micro-dystrophin replacement, which remain limited by transient effects, high cost, and restricted accessibility.

The recent clinical success of genome editing in monogenic disorders, such as sickle cell disease, provides strong validation for the translational potential of these approaches. In DMD, preclinical studies have already demonstrated substantial dystrophin restoration across multiple models, supporting continued progress toward clinical application.

However, key challenges—including efficient systemic delivery, long-term persistence of editing, and mitigation of immune and off-target effects—must be addressed to enable widespread clinical use. Ongoing advances in editing technologies, delivery platforms, and safety optimization are steadily overcoming these barriers.

Ultimately, the successful integration of these innovations may enable genome editing to evolve into a transformative, disease-modifying therapy capable of fundamentally altering the clinical course of DMD.

## Figures and Tables

**Table 1:** Available FDA-approved therapies for DMD.

Therapeutic Class	Drug (Generic/Brand)	Manufacturer	Mechanism of Action	Indication (FDA-Approved Population)	Year of FDA Approval	References
Corticosteroids (Standard of Care)	Prednisone/Prednisolone	Multiple manufacturers	Anti-inflammatory; slows muscle degeneration and prolongs ambulation	DMD (standard care)	21 February 1955	[91,92,93,94]
	Deflazacort (Emflaza)	PTC Therapeutics	Corticosteroid; reduces inflammation and muscle damage	Patients ≥ 2 years	9 February 2017 (expanded on 7 June 2019)	[91,93,94]
	Vamorolone (Agamree)	Santhera Pharmaceuticals	Dissociative steroid with anti-inflammatory activity and reduced glucocorticoid side effects	Patients ≥ 2 years	26 October 2023	[93,95,96,97,98]
Exon Skipping Antisense Oligonucleotides (Mutation-Specific)	Eteplirsen (Exondys 51)	Sarepta Therapeutics	Exon 51 skipping to restore dystrophin reading frame	~13% of DMD patients (exon 51 mutation)	19 September 2016	[99,100]
	Golodirsen (Vyondys 53)	Sarepta Therapeutics	Exon 53 skipping	~8% of DMD patients (exon 53 mutation)	12 December 2019	[101,102,103]
	Viltolarsen (Viltepso)	NS Pharma (Nippon Shinyaku)	Exon 53 skipping	~8% of DMD patients (exon 53 mutation)	12 August 2020	[104,105,106,107,108]
	Casimersen (Amondys 45)	Sarepta Therapeutics	Exon 45 skipping	~8% of DMD patients (exon 45 mutation)	25 February 2021	[109,110,111]
HDAC Inhibitor (Nonsteroidal Disease-Modifying Therapy)	Givinostat (Duvyzat)	Italfarmaco	Histone deacetylase (HDAC) inhibitor; reduces inflammation and fibrosis, delays progression	DMD patients ≥ 6 years	21 March 2024	[112,113,114]
Gene Therapy (AAV-Mediated Micro-Dystrophin)	Elevidys (delandistrogene moxeparvovec-rokl)	Sarepta Therapeutics	AAV vector delivering micro-dystrophin transgene	Mutation-independent therapy; contraindicated for DMD exon 8/9 deletions due to risk of severe immune-mediated myositis. Initially approved for ambulatory patients aged ≥4 years	22 June 2023 (expanded 20 June 2024)	[115,116,117,118]

## Data Availability

No new data were created or analyzed in this study.
